# Skin Bank Establishment in Treatment of Severe Burn Injuries: Overview and Experience with Skin Allografts at the Vienna Burn Center

**DOI:** 10.3390/jcm12144717

**Published:** 2023-07-17

**Authors:** Clement J. Staud, Annika Resch, Alexandra Christ, Anton Borger, Maximilian Zaussinger, Maryana Teufelsbauer, Nina Worel, Christine Radtke

**Affiliations:** 1 Department of Plastic, Reconstructive and Aesthetic Surgery, Medical University of Vienna, Spitalgasse 23, 1090 Vienna, Austria; 2 Department of Transfusion Medicine and Cell Therapy, Medical University of Vienna, Spitalgasse 23, 1090 Vienna, Austria

**Keywords:** skin allograft, skin bank, burn wound, skin regeneration, skin substitute

## Abstract

Depending on their extent, burn injuries require different treatment strategies. In cases of severe large-area trauma, the availability of vital skin for autografting is limited. Donor skin allografts are a well-established but rarely standardized option for temporary wound coverage. Ten patients were eligible for inclusion in this retrospective study. Overall, 202 donor skin grafts obtained from the in-house skin bank were applied in the Department of Plastic and Reconstructive and Aesthetic Surgery, Medical University of Vienna. Between 2017 and 2022, we analysed the results in patient treatment, the selection of skin donors, tissue procurement, tissue processing and storage of allografts, as well as the condition and morphology of the allografts before application. The average Abbreviated Burn Severity Index (ABSI) was 8.5 (range, 5–12), and the mean affected total body surface area (TBSA) was 46.1% (range, 20–80%). In total, allograft application was performed 14 times. In two cases, a total of eight allografts were removed due to local infection, accounting for 3.96% of skin grafts. Six patients survived the acute phase of treatment. Scanning electron microscope images and histology showed no signs of scaffold decomposition and intact tissue layers of the allografts. The skin banking program and the application of skin allografts at the Vienna Burn Center can be considered successful. In severe burn injuries, skin allografts provide time by serving as sufficient wound coverage after early necrosectomy. Having an in-house skin banking program at a dedicated burn centre is particularly advantageous since issues of availability and distribution can be minimized. Skin allografts provide a reliable treatment option in patients with extensive burn injuries.

## 1. Introduction

Burn injuries are among the most prevalent injuries, leading not only to a significant personal burden but also to an enormous economic burden, affecting almost 11 million people globally and causing more than 300,000 deaths per year [1,2]. The treatment of severe burn wounds requires tremendous financial resources, as well as intensive multidisciplinary management [3]. Intensive care capacity and resources are of particular importance and should be used with caution. Hence, prevention has been declared to be the easiest and most efficient means of burn treatment [3,4]. 

Extensive burn injuries are difficult to treat and mostly require autologous skin transplantation, which can be considered the gold standard for the replacement of damaged skin after burn trauma [5,6]. Procuring enough viable skin can be challenging after performing necrosectomy in patients with a large affected total body surface area (TBSA) [7]. Hence, temporary wound coverage, such as bioengineered skin substitutes, biological skin replacements and standard wound dressings, is necessary [8,9,10]. An important aspect of donor skin application is years of experience since skin allografting has been performed for decades [11]. It is essential to determine possible alternatives to skin allografting. There are numerous options available, either temporary dressings or permanent skin substitutes [12,13,14] (Table 1). 

Allografts are mainly used for temporary wound coverage when vital skin for procurement is limited after performing early necrosectomy for burn injuries affecting a large TBSA. In addition, donor skin grafts can be used for wound bed preparation and sandwich grafting techniques [9]. Recent studies have provided evidence that donor skin should not be strictly regarded as a form of temporary wound coverage, which has to be replaced within one to two weeks due to graft rejection [15,16]. It has been observed that allografts have survived permanently and acted as patients’ skin, showing no sign of rejection [15,16,17]. However, this outcome has only been reported anecdotally, and skin allografts are generally used as temporary coverage.

Availability is vital for any type of wound coverage. To ensure a reliable supply of allografts for use in the management of major burns, skin banks were established in many countries [18].

Cai et al. identified major tasks to create a sustainable skin banking program [19]:

- Identify and acquire the equipment and personnel needed to collect, process, store and graft cadaveric skin for burn injuries; and
- Develop safe donation protocols and documentation tools that remain feasible for low-resource settings.

With this article, we aim to summarize our experiences, learning and outcomes regarding donor skin grafts. Above all, we provide insights in the standard operation procedures (SOPs) at our skin banking program, which might be helpful for newly developing burn centres.

## 2. Materials and Methods

### 2.1. Allograft Program at the Department of Plastic, Reconstructive and Aesthetic Surgery: For the In-House Skin Banking Program, Standard Operating Procedures (SOP) Regarding the Procurement, Process and Application of Allografts Were Established

Procurement of skin: In cases of cadaveric organ donation, the plastic surgeon on duty is informed by the transplant coordinator. A protocol with a list of exclusion criteria has to be completed to verify whether a donor is suitable for skin procurement or not (Table 2).

**Figure 1:**
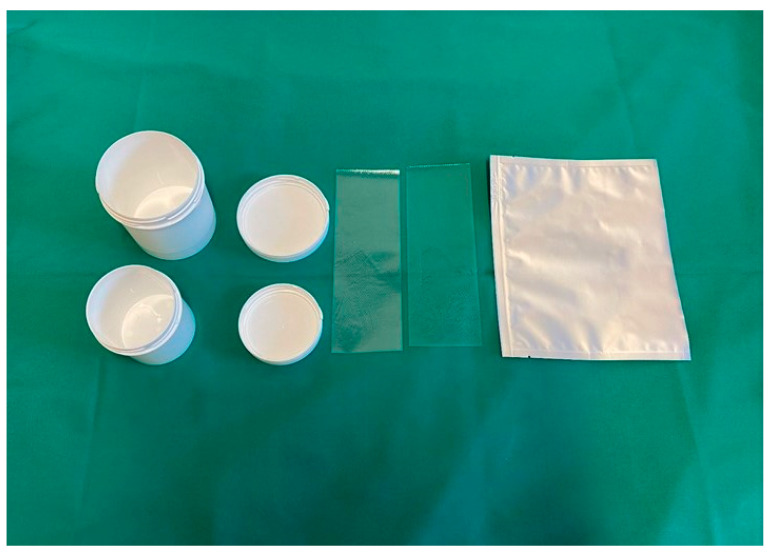
Sterile storage pots and bags for the transportation of skin allografts to the tissue establishment. The allografts are procured in the operating room and then labelled adequately to ensure traceability.

The plastic surgeon is then responsible for skin procurement and dispatch to the tissue establishment, which is in charge of skin processing and storage as part of the Department of Blood Group Serology and Transfusion Medicine. Procurement of viable skin is performed under sterile conditions in the operating theatre subsequent to the internal organs. The donor skin region is lubricated with fatty gauze, and afterward, the skin grafts (thickness between 0.4 and 0.6 mm, standard size 25 × 5 cm) are procured with a hydraulic dermatome. Subsequently, the skin is wrapped in sterile saline-soaked compresses, packed into storage pots that are labelled adequately to ensure traceability and sent to the tissue establishment (Figure 1).

Both procurement and processing facilities are accredited by the national competent authority according to the respective European Commission directives. In addition, the “Guide to the quality and safety of tissues and cells for human application”, published by the European Directorate for the Quality of Medicines and Healthcare (EDQM), provides guidelines on how donor evaluation, testing, procurement, transport, processing, labelling and storage are performed [20].

**Figure 2:**
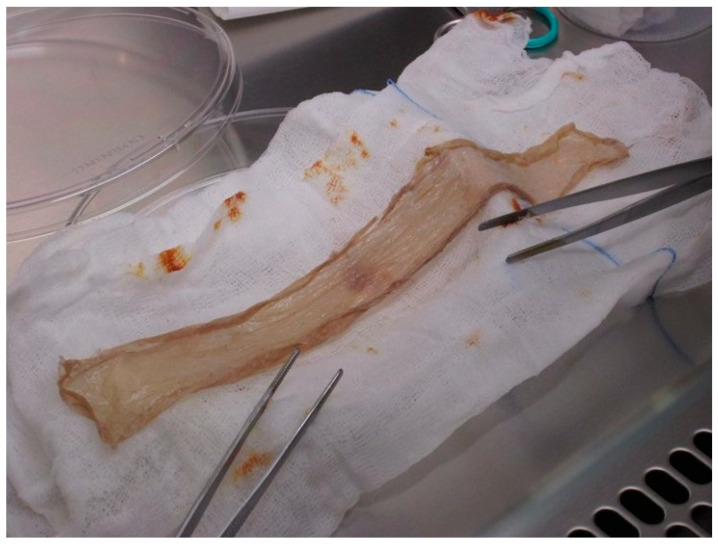
A skin allograft (thickness between 0.4 and 0.6 mm) in processing at the tissue establishment after removing the antiseptic povidone–iodine solution by washing it in saline twice.

Processing and storage of skin: At the tissue bank, the skin is soaked in povidone–iodine (Betaisodona^®^ Mundipharma, Austria) solution as a broad-spectrum antiseptic for 10 minutes and then washed in saline twice. Microbiological smears are obtained at the time of arrival and after decontamination, and both have to be negative, or the skin graft is discarded. As a next step, the skin is washed in saline for another 10 min to remove as much Betaisodona^®^ solution as possible (Figure 2).

Finally, the donor skin is packed in a sterile bag with dimethyl sulfoxide (10%) and tissue culture medium (TCM) 199 (90%) and then again packed in another non-sterile bag for storage, frozen in liquid nitrogen. The skin can be stored for up to five years. 

Application of skin: When a skin allograft is required, a notification is sent to the tissue bank. The skin grafts are delivered within one hour in nitrogen vapor shippers. The allografts then undergo a defrosting process which takes about 15 min. First, they are placed in ice water for 5 min and then brought up to ambient temperature. Further procedures are performed under sterile conditions. The skin is washed at least three times in saline to remove of any antifreeze solution and thus prepare it for application. The skin grafts are then meshed 1:1.5 or 1:3, put in place and fixed with staples before a bandage with one layer of fatty gauze and a dry cover-up dressing is applied. There is a protocol that has to be completed after the surgery. Adverse events with an impact regarding quality or safety of the donor skin or its application have to be logged and reported to the tissue bank. The data are subsequently saved digitally to allow for tracking of the donors, as well as the recipients’ profile for clinical reference and management. 

Wound dressing scheme: A bandage change is performed every second day: After removing the dressing, the wounds are treated with Prontosan^®^ (B. Braun, Vienna, Austria)-soaked compresses for 15 min. These compresses are then discarded, and a new bandage with one layer of fatty gauze and a dry cover-up dressing is applied accordingly. Wound swabs are routinely obtained once per week or more in cases of suspected infection.

### 2.2. Skin Allografts Analysed by Scanning Electron Microscope (SEM) and Histological Sectional Images

To investigate the condition and precise morphology of the grafts before application after processing and preparation, we analysed the tissue by SEM and histological section images. For preparation of the samples, an ascending alcohol series is applied for dehydration (10%, 20%, 30%, 50%, 70%, 90% and 3 × 100% (20 min each)), followed by chemical drying with hexametyldisilizane (HDMS) (Sigma-Aldrich, Darmstadt, Germany) for 45 min at ambient temperature. The sample is then dried for two hours under the laboratory fume hood. As a next step, the sample is fixed with double-sided tape to prepare it for gold sputtering with a Quorum Sputter Coater (Q 150 ES, Maidenhead, UK) using the program for primary sputtering with 40 nm of gold. The sample is then examined by electron microscopy using a Zeiss EVO 10 (Carl Zeiss GmbH, Wien, Austria). 

For the histological sectional images, the rehydration process of the samples is performed using a descended -OH series (Xylene, 95%, 95%, 70% and 50% (each for 3 min), followed by ddH_2_O for 5 min). The slides are incubated with haematoxylin for 3 min, then washed with tap water and rinsed in a chamber for 15 min. Then, eosin is added for 1 min, and afterwards, the samples are washed with ddH_2_O. Dehydration of the samples is performed using Xylene, 50%, 75% and 95% for 3 min each. Last, the samples are covered with mounting media and cover glasses to be ready for microscopy.

### 2.3. Patient Data and Evaluation of the Results

In total, 10 patients were eligible for inclusion in this retrospective study. Overall, 202 donor skin grafts obtained from the in-house skin bank at the Department of Plastic and Reconstructive and Aesthetic Surgery, Medical University of Vienna, were applied. Between 2017 and 2022, we analysed the outcomes in patient treatment, as well as the selection of skin donors, tissue procurement, tissue processing and storage of allografts. Clinical data were retrieved retrospectively using the routine clinical documentation system. The following parameters were collected: age, sex, Abbreviated Burn Severity Index (ABSI), TBSA, burn thickness, dates of admission to the intensive care unit (ICU) and discharge or death, cause of trauma, cause of death, number of surgeries, number of allografts applied, wound documentation and medical records.

## 3. Results

### 3.1. Patient Data and Outcome

**Figure 3:**
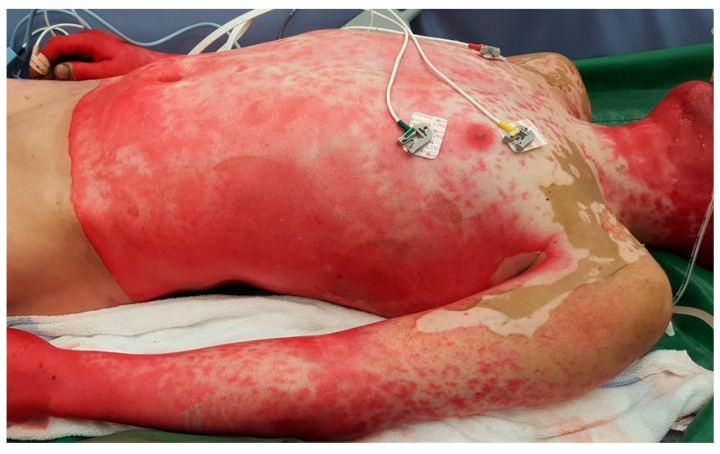
Grade III burn injury during the admission process to our burn unit. The patient suffered from a domestic fire, the injuries affected about 70% of TBSA, and the ABSI was 11. After cardiorespiratory stabilization, the burn wounds were cleaned of loose debris. As a next step, the wounds were treated with antiseptic solution before the bandaging was applied.

From 2017 to 2022, 10 patients were treated with 202 donor skin grafts (Table 3). Seven men and three women with an average age of 38.6 years old were enrolled (range, 18–68). The average ABSI score at the time of admission to the ICU was 8.5 (range, 5–12). The mean TBSA was 46.1% (range, 20–80%). The mean duration of inpatient treatment in the ICU was 54.4 days (range, 17–82). Six patients survived the acute phase of treatment, determined by discharge from the ICU. Three patients died due to multi-organ failure and one due to brain haemorrhage. The average number of surgeries was 5.9 (range, 4–11), including all surgeries performed in the acute phase of treatment in the ICU. The causes of injury were six accidents at work, two traffic accident, one scalding injury in the bathtub at home and one suicide attempt (Figure 3). 

**Figure 4:**
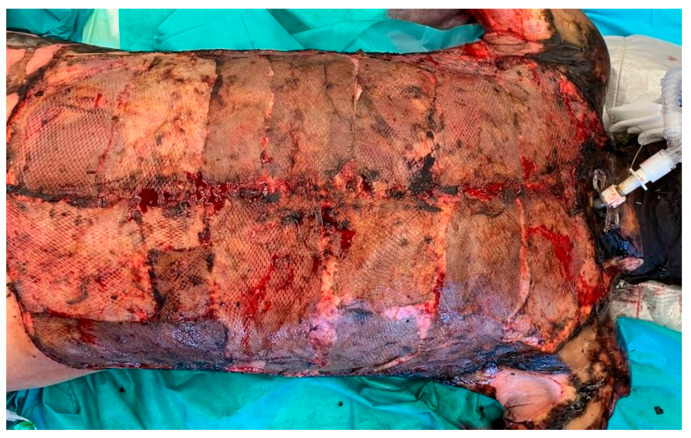
Severe grade III burns of the upper body after early necrosectomy was performed. The wounds are covered with skin allografts. On day eight after surgery, the grafts were in place and served as wound coverage. There were no clinical signs of infection. Smears at the time were negative.

Initial early necrosectomy was performed in all patients. Vital skin was harvested for autograft transplantation if the patients were cardiorespiratory stable. The remaining wounds were covered with allografts. (Figure 4). 

**Figure 5:**
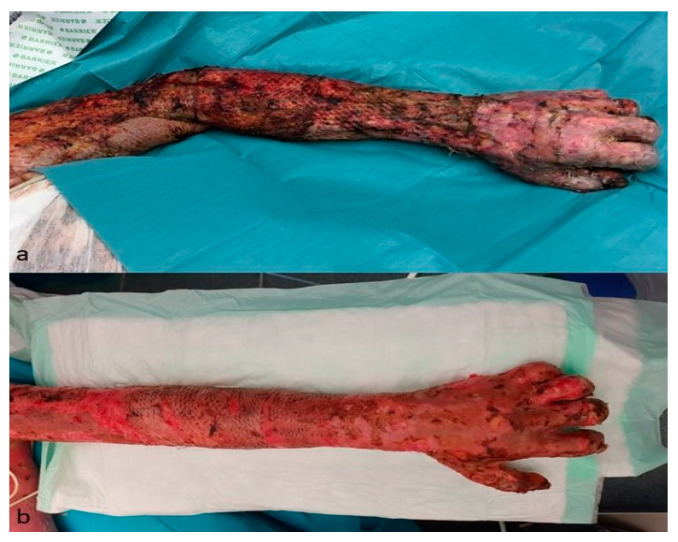
Grade III burn injury of the left upper extremity. (**a**)—Five days after necrosectomy and covered by meshed allograft skin (both hands were covered with unmeshed autograft). (**b**)—Three weeks after removal of the allograft and replacement by autografts.

**Figure 6:**
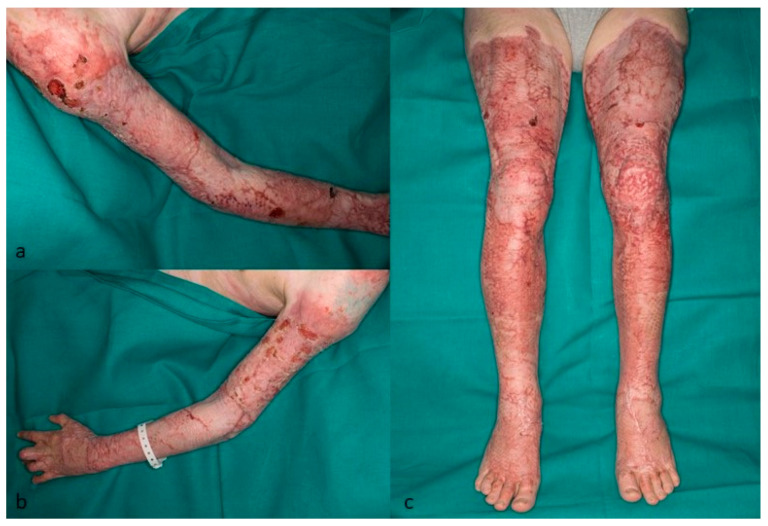
(**a**–**c**) Five months after skin autograft transplantation. The patient still had to wear compression garments. There were only a few skin lesions left.

The skin allografts served as wound coverage for 12–14 days and were then removed and replaced by autologous skin grafts (Figure 5a,b and Figure 6a–c). 

In all cases the allografts were replaced by autografts as soon as the patients were stable, and the donor sites for skin autografts were suitable for harvesting again. Two patients underwent skin allografting two times and one patient three consecutive times due to extraordinarily large affected TBSAs of 80%. Overall allograft-application was performed 14 times. In two patients, we observed signs of an infection in a total of eight allografts, which then had to be removed. This rate accounts for 3.96% of 202 skin grafts. The smears of one patient showed *Pseudomonas aeruginosa*, and the second patient had an infection caused by *Enterobacter cloacae*. Antibiotic treatment was established following an antibiogram, as well as a resistogram. The smears taken from the allografts at the time of application were negative. There were no signs of acute adverse events, such as allergic reactions. Five patients additionally had to receive a supplementary therapy for smoke inhalation. Furthermore, five out 10 patients were treated in a fluid air bed due to posterior surface injury. In four cases, the patients suffered acute renal failure, which required temporary renal replacement therapy. All four had lethal outcomes. 

### 3.2. Scanning Electron Microscope and Histology

**Figure 7:**
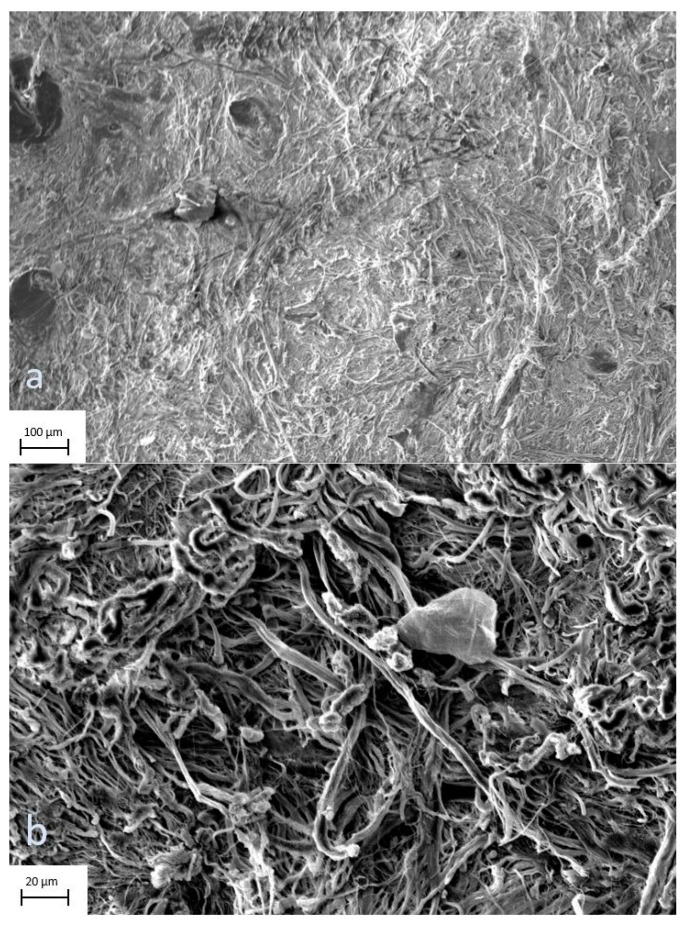
(**a**,**b**) Skin allografts on scanning electron microscopy. Profound views of the graft at 177× and 1000× magnification. There are no signs of scaffold decomposition after processing and preparation.

**Figure 8:**
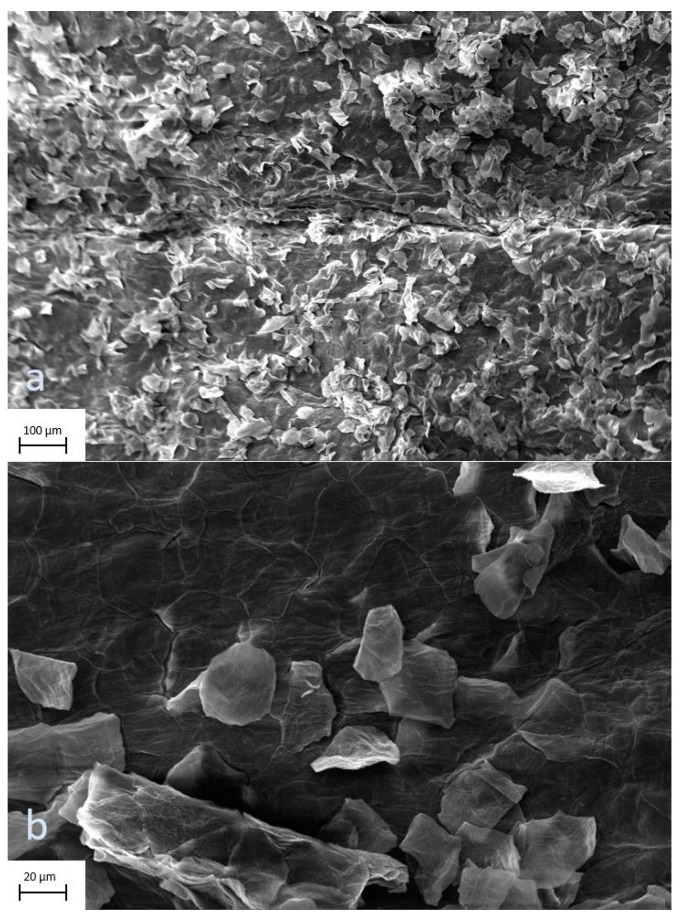
(**a**,**b**) Skin allografts on scanning electron microscopy. The epidermal view of the graft at 209× and 1000× magnification displays undisturbed continuity of the tissue.

The digital images of surface scans by electron microscope showed no signs of scaffold decomposition after processing and preparation. The scans of the profound side of the allograft at 177× and 1000× magnification display undisturbed continuity of the tissue (Figure 7a,b), as do the epidermal views at 209× and 1000× magnification (Figure 8a,b).

**Figure 9:**
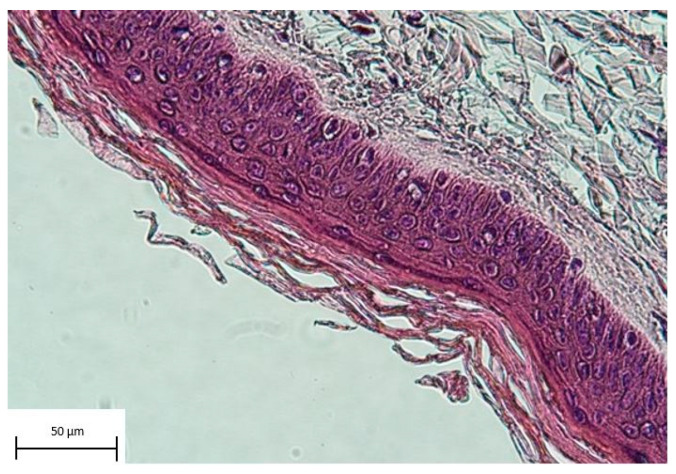
Histological section of an allograft stained with haematoxylin and eosin. The picture shows intact layers of epidermal tissue.

Histological sectional images stained with haematoxylin and eosin (HE) showed intact layers of epidermal tissue (Figure 9).

### 3.3. Skin Banking Program—Skin Donors, Procurement, Processing, Storage and Application of Allografts

During the observation period, 21 organ donors were suitable for skin procurement. The cause of death was in all cases severe haemorrhagic or hypoxic brain damage. The skin was harvested after the internal organs since multi-organ donation was carried out in all cases, following the protocol as described in the SOPs. There were no donations after circulatory death (DCDs). Processing and storage, as well as application, were performed accordingly. The smears of five donor skin grafts were positive for staphylococci at the time of processing and were therefore discarded. There were no further adverse medical or administrative events reported. In 14 consecutive cases of donor skin graft application, a total of 202 grafts were used.

## 4. Discussion

Early excision of the eschar is described as highly beneficial in treatment of burn injuries with large TBSAs [21,22,23]. Since unaffected skin areas for procurement of autografts are limited in this patient collective, it is vital to provide a source for expedient wound coverage. With a total of 3.96% (8 of 202) of allografts needing to be removed early because of infection, our findings show that skin allografts serve as a reliable temporary substitute. Alternatively, Ziegler et al. showed that a wide range of different treatment options, including biosynthetic skin substitutes, are used in burn centres across Europe. To provide optimal conditions for non-delayed wound healing, the evaluation showed polyhexanide to be the most common anti-microbiological topical agent, used in 83% of burn centres. Bromelain-based enzymatic debridement is performed in 18% of evaluated burn centres [24]. Another study by Carsin et al. showed that cultured epithelial autografts were applied successfully to provide permanent coverage in severely traumatized patients, which has to be considered when using temporary substitutes [25].

There are various features that ideal wound coverage should provide. The ultimate material is widely available, durable and flexible; withstands shear forces, is inexpensive, and nonantigenic; prevents water loss; provides a barrier to bacteria; and conforms to irregular surfaces. Skin substitutes can be divided into two main groups: biological and synthetical—both have special characteristics and therefore advantages and disadvantages. Biological substitutes provide a more natural new dermis and better reepithelialization, whereas synthetical substitutes ensure advantages in scaffold composition [13]. Our results of the surface scans by electron microscope, as well as the histological sectional images, displayed no signs of scaffold decomposition and intact layers of tissue, comparable to samples of fresh skin [26,27]. This finding indicates that processing does not affect the grafts negatively, which is noteworthy since it has been reported that human leukocyte antigen (HLA)-matched allografts were successfully applied and showed long-term persistence without rejection [15,16]. 

Donor skin allografts are a well-established, but rarely standardized, option for temporary wound coverage. There are various aspects regarding the application of skin allografts and initiating a skin banking program. Although there has been a tendency to establish specialized burn units worldwide [11], there are still many regions or even countries without adequate medical care for burn victims [28]. In addition to potential individual hurdles, wound coverage after necrosectomy—especially after large area trauma—is an issue for every burn centre. Unlike other standard wound dressings, which can involve dependence on a company or supplier, donor skin allografts can be procured, stored and applied regardless of specific supply, as the required materials for skin banking can be obtained from various sources. 

Since there is also a temporal aspect of the treatment of burn injuries, it is a matter of timely availability. At our burn unit, skin allografts are available at our skin bank with about one hour of lead time. The allograft skin can be stored in frozen liquid nitrogen for up to five years, simplifying the perpetual maintenance of sufficient supply. We believe that it is very beneficial to have easy access to skin allografts, preferably on an in-house or national basis because it is more flexible than obtaining allografts from international tissue banks, which includes dependency, comparable to special dressings.

To implement a skin banking program and to achieve a high level of quality and patient safety, legal requirements have to be fulfilled. In our department, we have pursued that aim by constant development and staff training, guaranteed through process validation by the national competent authority, AGES (The Austrian Agency for Health and Food Safety, Vienna, Austria). In addition to all medical considerations, there might be cultural and religious issues as well since this treatment includes applying tissue from cadavers [19,29]. To overcome those barriers, it is necessary to spread awareness, and education is key to doing so. In numerous countries, the concept of organ donation is practically unknown. There is a strict legal framework in Austria concerning organ donation. It basically states that everyone is an organ donor if there no dissent has been reported during the lifetime, also known as an opt-out system. Therefore, the organ transplant activity is comparatively high [30].

Today, despite constant research and evolution, there is no ideal wound dressing or skin substitute that fulfils all medical demands without risks or disadvantages [31]. Even for dedicated burn units, the treatment of patients with large affected body surface areas is challenging. The application of skin allografts can contribute to reducing mortality [21,23]. The choice of the best treatment still has to be made individually for each patient.

## 5. Conclusions

The skin banking program and the application of skin allografts at the Vienna Burn Center can be considered successful. In severe burn injuries, skin allografts provide time by serving as sufficient wound coverage after early necrosectomy. Having an in-house skin banking program at a dedicated burn centre is particularly advantageous since issues of availability and distribution can be minimized. We can state that skin allografts serve as reliable temporary coverage to allow for necrosectomy and stabilization before definitive coverage with autologous skin grafts in patient with extensive burn injuries.

## Figures and Tables

**Table 1:** Examples of temporary dressings and permanent skin substitutes that can be used in burn treatment for wound coverage.

Temporary Dressings	Dermal Substitutes	Epidermal Substitutes
Allografts	Matriderm^®^	ReCell^®^
Xenografts	Integra^®^	Epicel^®^
Amnion	Renoskin^®^	Laserskin^®^
Acticoat^®^	Pelnac^®^	Vivoderm^®^
Aquacel AG^®^	Dermagraft^®^	EpiDex^®^
Biobrane^®^	Alloderm^®^	TranSCell^®^

**Table 2:** List of exclusion criteria for allograft procurement. If one of the listed points, applies the patient is not eligible for donor skin harvesting.

✓ Unknown cause of death ✓ Disease of unknown aetiology ✓ Presence or history of malignant disease ✓ Risk of disease transmission by prions: - Creutzfeldt–Jakob disease - Individuals with a history of rapidly progressive dementia or degenerative neurologic disease ✓ Active (systemic) infections ✓ Connective tissue diseases ✓ Recipients of xenografts ✓ Chemotherapy and/or immunosuppressant use (if less than three months before death)	✓ Signs for risk of transmission of HIV, HBV, HCV, HTLV or other infectious diseases ✓ Physical signs on the donor’s body suggesting a risk of infectious disease ✓ Confirmed or suspected vaccination with live vaccine within the previous four weeks, with risk of transmission considered possible ✓ Extraneous history of chronic, systemic autoimmune disease ✓ Signs of invalid test results from donor blood samples ✓ Myocarditis or endocarditis

**Table 3:** Summarized data of all 10 patients treated with allograft skin.

Patient	Age	Sex	Days in ICU	Burn Thickness	TBSA (%)	Cause of Trauma	ABSI	Death	Number of Surgeries	Number of Allografts
1	50	m	41	3	30	Work	7	yes	5	11
2	68	w	39	2	30	Traffic	8	no	4	4
3	39	w	17	3	20	Suicidal	6	no	6	8
4	29	m	53	3	70	Work	11	no	4	69
5	27	m	58	3	15	Traffic	5	no	5	21
6	17	m	82	2	51	Work	7	no	5	24
7	34	m	40	3	60	Work	10	yes	4	40
8	41	m	63	3	55	Leisure	9	no	6	5
9	32	m	80	3	70	Work	12	yes	11	15
10	49	m	71	3	60	Work	10	yes	10	5
*Median*	*36.5*		*55.5*		*53.0*		*8.5*		*5.0*	*13.0*

## Data Availability

The data presented in this study are available on request from the corresponding author.
